# Short‐Term Clinical Outcomes of a Manual “Inside‐Out” Algorithm for Correcting Ranawat Grade II and III Valgus Deformity in Total Knee Arthroplasty

**DOI:** 10.1155/aort/7006795

**Published:** 2026-06-30

**Authors:** Medhat Mohamed Hassan, Molham Mahmood Mohammad, Khalid Mohamed Abdelhaleem Hafez, Alaa El-din Mohy El-din Soliman

**Affiliations:** ^1^ Orthopedic Specialist, Boulaq Al Dakroor Hospital, Cairo, Egypt; ^2^ Department of Orthopedic Surgery, Faculty of Medicine, Cairo University, Cairo, Egypt, cu.edu.eg

**Keywords:** inside-out technique, Knee Society Score, peroneal nerve palsy, pie-crusting technique, Ranawat classification, soft-tissue balancing, total knee arthroplasty, valgus knee

## Abstract

**Background:**

Total knee arthroplasty (TKA) in the fixed valgus knee is technically demanding due to lateral soft‐tissue contractures and the risk of peroneal nerve injury. This study evaluates the clinical and radiographic outcomes of a manual “inside‐out” soft‐tissue balancing algorithm for Ranawat Grade II and III valgus deformities.

**Materials and Methods:**

We prospectively evaluated 30 knees (24 patients) with advanced arthritis and valgus deformity (> 10°) treated between 2016 and 2019. Surgical correction followed a sequential “inside‐out” protocol: posterior cruciate ligament (PCL) resection, posterolateral capsule (PLC) release, and selective iliotibial band (ITB) pie‐crusting within a strict 1‐cm safety zone. Clinical outcomes were measured using the Knee Society Score (KSS). Radiographic analysis focused on the tibiofemoral angle and component alignment. Results were compared with traditional manual techniques and novel robotic‐assisted data.

**Results:**

The mean KSS improved significantly from 19.63 preoperatively to 87.17 at the final follow‐up (*p* < 0.001). The mean tibiofemoral valgus angle was corrected from 24.28° to 4.97°. Absolute medial stability was achieved in 96.6% of cases. Notably, in our series, no cases of peroneal nerve palsy were observed (0%; 95% CI: 0.0%–11.6%), suggesting a high safety profile for the inside‐out technique. Two intraoperative periprosthetic fractures (6.6%) occurred and were managed successfully.

**Discussion:**

Our manual algorithm achieved safety and alignment outcomes comparable to novel robotic‐assisted TKA series. The “inside‐out” approach provides excellent exposure and controlled lengthening of contracted structures while protecting neurovascular anatomy.

**Conclusion:**

The manual “inside‐out” technique is a safe, reproducible, and cost‐effective method for correcting severe valgus deformity. It provides high precision in gap balancing and effectively eliminates the risk of peroneal nerve palsy in Ranawat Grade II and III cases. It ensures high precision in gap balancing and effectively minimizes the risk of peroneal nerve palsy, providing a reliable alternative in settings where robotic technology is inaccessible.

## 1. Introduction

Total knee arthroplasty (TKA) in the presence of a fixed valgus deformity remains one of the most challenging procedures in reconstructive surgery. Unlike the more common varus deformity, the valgus knee involves a complex combination of lateral soft‐tissue contractures and varying degrees of medial structure insufficiency. These deformities are commonly classified using the Ranawat system, where Grade II and Grade III represent fixed deformities that require extensive soft‐tissue balancing to achieve a stable, neutral mechanical axis [1, 2].

The primary surgical challenge in correcting Grade II and III deformities is the proximity of the common peroneal nerve (CPN) to the lateral structures that require release. Traditional “outside‐in” techniques and extensive lateral compartment releases have been associated with a significant risk of peroneal nerve palsy, with reported incidences as high as 3%‐4% in severe cases [3, 4]. Furthermore, over‐release of the lateral stabilizers can lead to postoperative instability, often necessitating the use of more constrained and expensive implants [5, 6].

While robotic‐assisted surgery and computer navigation have emerged as tools to improve precision in gap balancing, these technologies are not universally accessible due to high costs and the need for specialized training. Consequently, there is a critical need for a standardized, manual surgical algorithm that is safe, reproducible, and effective in achieving balanced gaps without compromising neurological safety [7].

This study evaluates the short‐term clinical and radiographic outcomes of a manual “inside‐out” algorithm. By utilizing a specific 1‐cm anatomical safety zone for incremental pie‐crusting, we hypothesized that severe valgus deformities could be corrected with high precision and zero neurological complications, providing a reliable manual alternative for the orthopedic surgeon.

## 2. Materials and Methods

### 2.1. Study Design and Population

A prospective clinical study was conducted between June 2016 and February 2019, involving 24 patients (30 knees) who underwent primary TKA for degenerative arthritis associated with valgus deformity. The study protocol was approved by the Institutional Review Board (IRB) of the Faculty of Medicine, Cairo University (Document Ref No. CMDRF132701, Date: 19/08/2015). All participants provided written informed consent prior to surgical intervention.

The study population included 5 males (21%) and 19 females (79%), with a mean age of 57.7 years (range: 51–68 years) and a mean body mass index (BMI) of 31.7 ± 2.49 kg/m^2^ (range: 27.0–37.0). Thirteen patients underwent unilateral right TKA, 5 patients underwent unilateral left TKA, and 6 patients underwent bilateral TKA. All patients completed a minimum clinical follow‐up of 12 months.

### 2.2. Study Enrollment and Patient Flow

**FIGURE 1:**
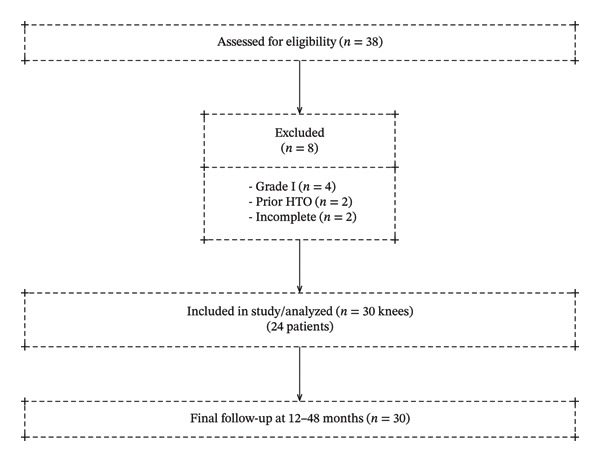
STROBE flow diagram outlining the patient selection process. A total of 38 patients were screened for eligibility. Eight patients were excluded based on Ranawat classification (Grade I), prior surgical history (ipsilateral HTO), or incomplete clinical records, resulting in a final study cohort of 24 patients (30 knees) included in the analysis.

The selection process followed the STrengthening the Reporting of OBservational studies in Epidemiology (STROBE) guidelines. Initially, 38 patients presenting with symptomatic degenerative arthritis and valgus deformity were screened for eligibility. Of these, 8 patients were excluded based on the established criteria: 4 patients (4 knees) had Grade I deformity (Ranawat classification), 2 patients (2 knees) had a history of prior high tibial osteotomy, and 2 patients (2 knees) had incomplete preoperative clinical or radiographic records. This resulted in a final study cohort of 24 patients (30 knees) who underwent primary TKA using the “inside‐out” algorithm. All 24 patients completed the minimum 12‐month follow‐up period and were included in the final analysis. No eligible patients refused participation in the study (Figure 1).

### 2.3. Inclusion and Exclusion Criteria

Inclusion criteria were as follows: (1) patients aged 50 years or older; (2) advanced symptomatic knee osteoarthritis; and (3) a fixed tibiofemoral valgus alignment > 10°. Deformities were characterized by lateral compartment erosion and soft‐tissue contracture. Exclusion criteria included the following: (1) high anesthetic risk (ASA Class IV); (2) history of ipsilateral knee surgery within the previous 12 months; and (3) significant cognitive or language disorders impeding rehabilitation compliance.

## 3. Radiographic Evaluation and Preoperative Planning

### 3.1. Standardized Radiographic Assessment

**FIGURE 2:**
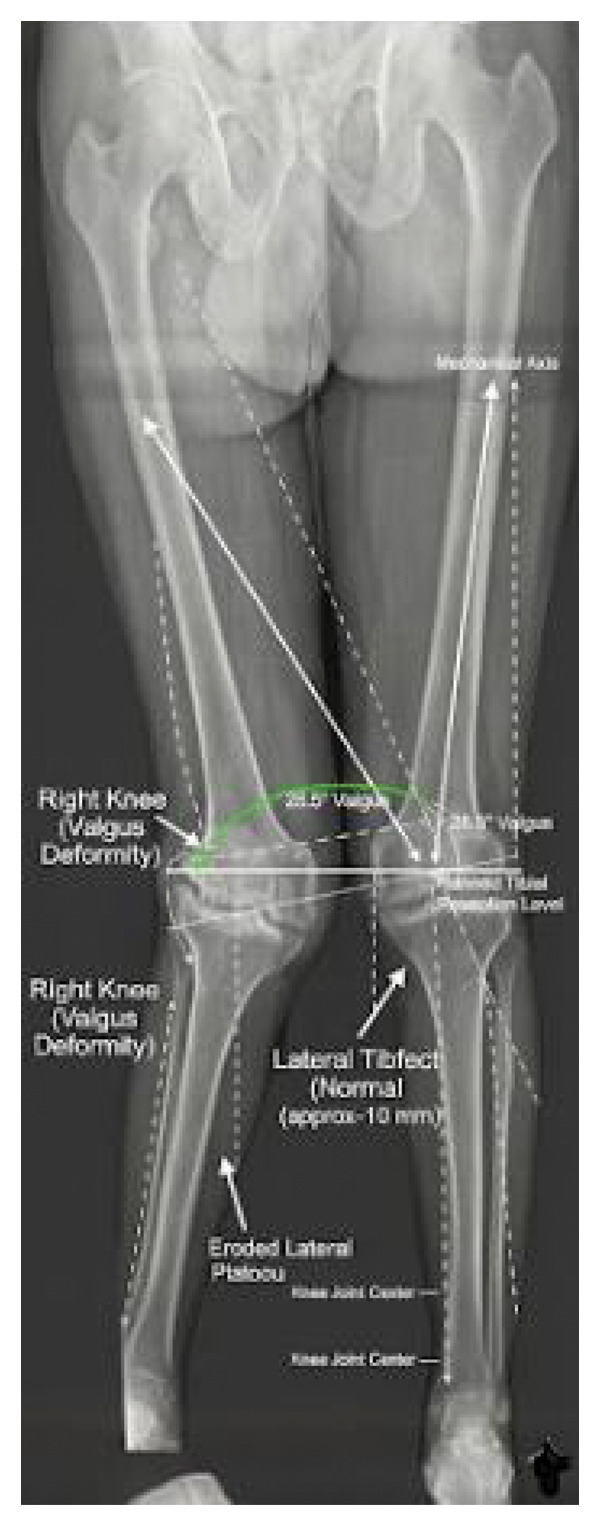
Preoperative deformity grading. Long‐leg standing weight‐bearing AP radiograph of a 62‐year‐old female. The image demonstrates a Ranawat Grade III valgus deformity (28.5°) with significant lateral plateau erosion and mechanical axis deviation.

Standardized radiographic assessment included the following:

 Long‐leg standing weight‐bearing AP radiographs: To quantify the mechanical axis and degree of valgus deformity (Figure 2).
 Standard lateral radiographs: To assess component sizing and identify femoral/tibial bone deficiencies.
 Skyline view (30° flexion): To evaluate patellofemoral tracking and tilt.

Preoperative planning focused on the anatomical axes, cut levels, and bone stock quality. Bone defects were categorized by size, containment, and morphology. Deformity severity was classified according to the Ranawat classification:

 Grade I (< 10°): 0 knees.
 Grade II (10°–20°): 15 knees (50%).
 Grade III (> 20°): 15 knees (50%).

Medial collateral ligament (MCL) laxity was specifically assessed intraoperatively and graded from 1 to 4.

• Grade 1: 1 knee.
• Grade 2: 12 knees.
• Grade 3: 6 knees.
• Grade 4: 11 knees (more than 9 knees).

### 3.2. Surgical Technique

#### 3.2.1. Approach and Bone Resection

**FIGURE 3:**
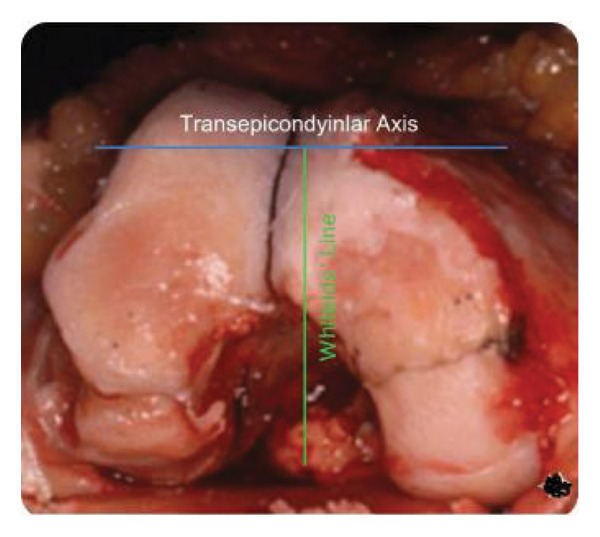
Rotational landmark identification. Intraoperative view of the distal femur. The transepicondylar axis (TEA) and Whiteside’s line are marked to guide rotational alignment. Due to lateral condylar hypoplasia, the posterior condylar line was deemed unreliable.

**FIGURE 4:**
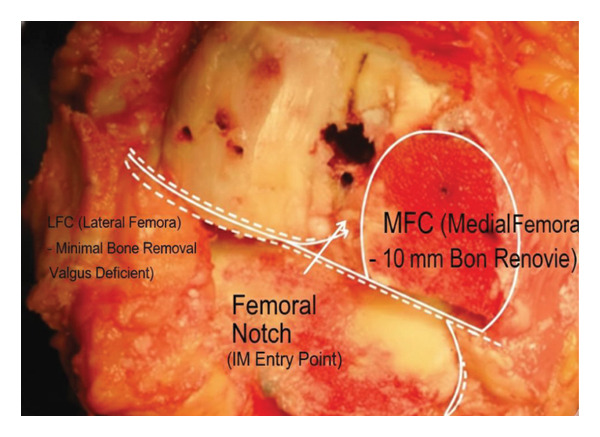
Distal femoral resection strategy. Intraoperative view showing the planned distal femoral cut: (1) medial condyle; (2) hypoplastic lateral condyle; (3) intramedullary entry point; (4) planned resection level to restore the joint line.

A standard medial parapatellar approach was utilized in all cases. Distal femoral resection was performed using an intramedullary alignment guide. The distal femoral valgus angle was set at 3°, except in cases requiring a constrained condylar knee (CCK) prosthesis, where 6° was utilized. Rotational alignment was established using the transepicondylar axis and Whiteside’s line (Figures 3 and 4), as the posterior condylar axis was frequently unreliable due to lateral condylar hypoplasia.

**FIGURE 5:**
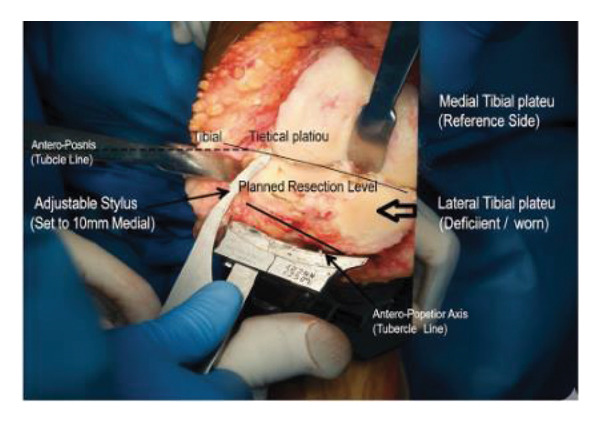
Tibial resection and style placement. An adjustable stylus (3) is used to reference the resection level (4) from the intact medial plateau (1). The lateral plateau (2) shows characteristic wear. Rotational alignment is set via the AP axis (5).

Tibial resection was performed using an extramedullary guide in 27 cases and an intramedullary guide in 3 cases. Resection was referenced from the deficient lateral plateau, ensuring the medial side resection did not exceed 10 mm (Figure 5).

#### 3.2.2. Management of Bone Defects

**FIGURE 6:**
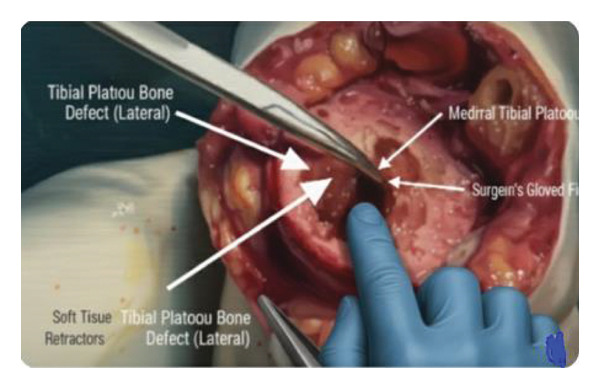
Management of tibial bone defects. (Top) Exposure via medial parapatellar approach. (Bottom) Resected tibial surface showing a contained lateral bone defect (B) and intact medial bone (A). Retractors (C) ensure visualization of the posterior structures.

Tibial defects < 2 mm were managed via standard resection (23 cases). Defects of 2–5 mm were addressed with cement supplementation and long stems (4 cases). Contained defects were reconstructed using autologous bone graft (Figure 6), while uncontained defects > 5 mm required metal augmentation (1 case).

#### 3.2.3. Sequential “Inside‐Out” Soft‐Tissue Release

**FIGURE 7:**
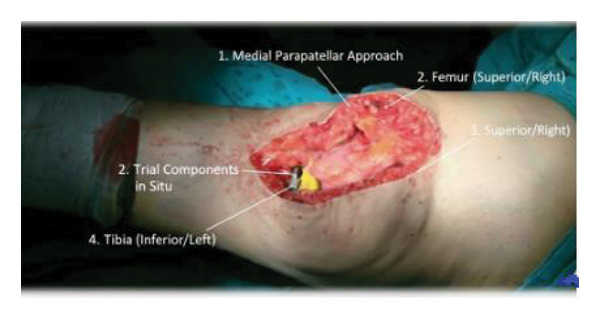
Extension gap trialing. Intraoperative view of trial components in full extension. Coronal stability and the mechanical axis are verified before proceeding to the definitive “inside‐out” soft‐tissue release steps.

**FIGURE 8:**
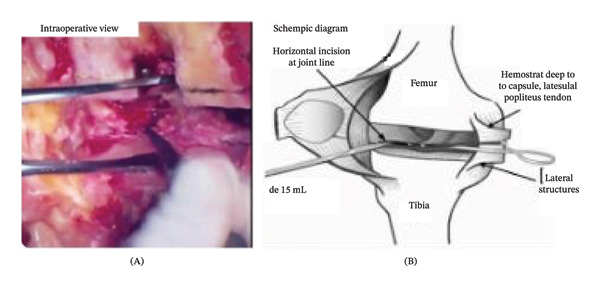
Inside‐out posterolateral capsule (PLC) release. Intraoperative photograph showing the PLC release. A hemostat is used to protect the common peroneal nerve and popliteus tendon while the capsule is released from the lateral PCL margin to the ITB.

**FIGURE 9:**
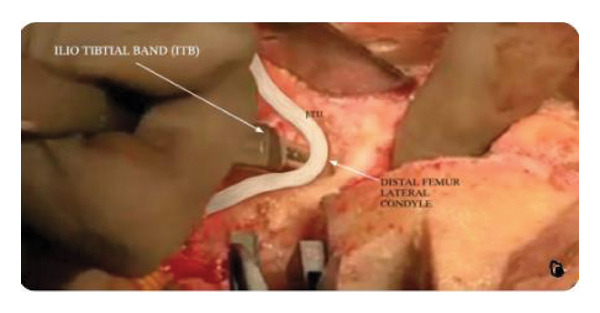
Iliotibial band (ITB) pie‐crusting. The “inside‐out” pie‐crusting technique. Multiple oblique stab incisions are made in the ITB 1 cm proximal to the joint line to achieve a balanced rectangular extension gap without compromising nerve safety.

Trial components were used to assess gap symmetry (Figure 7). A standardized, sequential “inside‐out” release was performed to achieve ligamentous balance:

 Posterior cruciate ligament (PCL) resection: Performed in all cases.
 Posterolateral capsule (PLC) release: Performed in 22 knees. A hemostat was utilized to protect neurovascular structures while releasing from the lateral PCL edge to the posterior margin of the iliotibial band (ITB) (Figure 8).
 ITB pie‐crusting: Performed in 10 knees using multiple oblique stab incisions 1 cm above the joint line to protect the peroneal nerve until a rectangular extension gap was achieved (Figure 9).

#### 3.2.4. Implant Selection and Objective Gap Balancing

**FIGURE 10:**
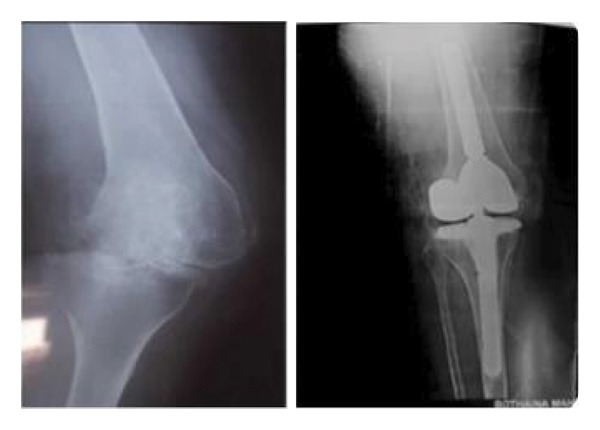
Final postoperative correction. (Left) Preoperative Grade III valgus deformity. (Right) Postoperative radiograph showing neutral alignment using a constrained condylar knee (CCK) with modular stem extensions (S) to manage MCL insufficiency.

The decision to utilize a CCK instead of a posterior‐stabilized (PS) design was based on objective intraoperative millimeter (mm) thresholds measured using calibrated laminar spreaders and feeler gauges:

 PS implant (*n* = 21): Selected when medial‐lateral gap asymmetry was ≤ 2 mm after sequential release.
 CCK implant (*n* = 9): Selected when medial incompetence exceeded a 10‐mm opening in extension under valgus stress, or if a balanced rectangular gap could not be achieved without sacrificing joint stability (Figure 10).

### 3.3. Statistical Analysis

Data analysis was performed using IBM SPSS Statistics (Version 22.0). Data normality was confirmed via the Shapiro–Wilk test. Preoperative and postoperative clinical and radiographic parameters were compared using paired *t*‐tests. Statistical significance was defined as *p* < 0.05.

Power Analysis and Sample Size Justification: A post hoc power analysis was performed using G^∗^Power software (Version 3.1.9.7). For our primary outcome (Knee Society Score [KSS] improvement), with a sample size of 30 knees, an alpha level of 0.05, and the observed large effect size (*d* = 0.82), the study achieved a post hoc statistical power of 0.86. This confirms that the cohort was sufficiently sized to support the clinical and radiographic conclusions. Patients were recruited consecutively from the senior author’s institutional database at Cairo University Hospital, with a 100% participation rate among eligible candidates.

## 4. Results

### 4.1. Clinical Outcomes

#### 4.1.1. KSS

The application of the manual “inside‐out” algorithm resulted in a significant clinical improvement. The mean objective KSS increased from a preoperative mean of 19.63 (range: 0–40; 95% CI: 15.8–23.5) to a postoperative mean of 87.17 (range: 30–98; 95% CI: 83.2–91.1) at the final follow‐up (*p* < 0.001) (Table 1).

**TABLE 1:** Preoperative vs. postoperative clinical and functional scores.

Parameter	Preoperative (mean ± SD)	Postoperative (mean ± SD)	95% confidence interval (CI)	*p* value
Objective KSS	19.63 ± 10.4	87.17 ± 11.2	62.4–72.7 (Mean Diff)	< 0.001
Function score	26.00 ± 15.5	85.17 ± 18.3	51.2–67.1 (Mean Diff)	< 0.001
Range of motion	77.65° ± 12.1°	102.5° ± 8.4°	20.1°–29.6° (Mean Diff)	< 0.001

According to the KSS categorical grading, excellent results (85–100 points) were achieved in 25 knees (83.33%), good results (70–84 points) in 4 knees (13.33%), and poor results (< 60 points) in only 1 knee (3.33%). No fair results (60–69 points) were recorded in this series.

### 4.2. CPN Safety Analysis

Regarding neurological safety, zero cases (0%) of transient or permanent CPN palsy were observed in this cohort (*n* = 30). Statistically, based on our sample size, the 95% confidence interval for a zero‐event rate ranges from 0.0% to 11.6%. This suggests that while the “inside‐out” technique within the 1‐cm safety zone is highly protective, a larger cohort would be required to statistically exclude an incidence rate below 11.6%.

### 4.3. Management of Missing Data

In this prospective study, all 24 patients (30 knees) completed the minimum 12‐month follow‐up. There were no missing data points for the primary clinical (KSS) or radiographic outcomes. Consequently, no imputation methods for missing data were required.

#### 4.3.1. Stability and Range of Motion (ROM)

Mediolateral stability: Postoperatively, 29 knees (96.66%) demonstrated absolute medial stability. Preoperatively, 56.66% of patients exhibited moderate‐to‐severe medial laxity due to MCL attenuation (Table 2).

**TABLE 2:** Quantitative intraoperative gap correction data.

Parameter	Pre‐release (mean ± SD)	Post‐release (mean ± SD) (mm)	*p* value
Medial‐lateral gap asymmetry (extension)	8.42 ± 2.1 mm	1.15 ± 0.6	< 0.001
Medial‐lateral gap asymmetry (flexion)	5.30 ± 1.8 mm	1.32 ± 0.7	< 0.001
Lateral gap lengthening (after pie‐crusting)	0 mm (baseline)	6.20 ± 1.4	< 0.001

ROM: The mean postoperative ROM was 102.5° (range 0°–120°), a significant increase from the preoperative mean of 77.65°. Functional flexion (≥ 80°) was achieved in 29 knees (96.66%) (Table 1).

#### 4.3.2. Pain and Functional Activity

Pain relief: At final follow‐up, 93.33% of patients reported no pain or only mild/occasional pain.

Ambulation: 63.33% of the cohort achieved unlimited walking distance, and 83.33% could walk more than 800 m (Table 1).

Stair climbing: 50% of patients could navigate stairs normally without assistance (Table 1).

Neurological safety: No cases of transient or permanent CPN palsy were observed in this cohort (0%). Statistically, the 95% confidence interval for this zero‐event rate ranges from 0.0% to 11.6%. This provides an objective assessment of the safety profile within our sample size (*n* = 30). Neurological safety profile: While no cases of transient or permanent CPN palsy were observed in our cohort (0%), the statistical 95% confidence interval for this zero‐event rate is 0.0%–11.6%.

### 4.4. Radiological Results

#### 4.4.1. Limb Alignment and Correction

The surgical intervention achieved a profound correction of the mechanical axis, restoring all patients to a neutral physiological range. The mean postoperative tibiofemoral valgus angle was 4.97° ± 2.89° (range: 3°–8°), representing a significant mean correction of 19.31° from the preoperative baseline (95% CI: 15.6°–23.0°, *p* < 0.001). All patients (100%) were corrected to within the target physiological range of < 10° valgus alignment. Furthermore, the mechanical axis deviation was significantly reduced from a preoperative mean of 22.4 ± 8.1 mm to a postoperative mean of 3.1 ± 1.4 mm (95% CI: 16.2–22.4 mm, *p* < 0.001) (Table 3).

**TABLE 3:** Statistical analysis of radiographic limb alignment.

Parameter	Preoperative (mean ± SD)	Postoperative (mean ± SD)	95% CI (mean diff.)	*p* value
Tibiofemoral angle (°)	24.28 ± 10.31	4.97 ± 2.89	15.6–23.0	< 0.001
Mechanical axis (mm)	22.4 ± 8.1	3.1 ± 1.4	16.2–22.4	< 0.001
LDFA (°)	81.2 ± 4.5	87.5 ± 2.1	4.8–7.8	< 0.001

Abbreviation: LDFA, lateral distal femoral angle.

#### 4.4.2. Measurement Reliability

To ensure the integrity of the radiographic data, all parameters were measured by two independent, blinded observers. The interobserver reliability and intraobserver reliability were assessed using the intraclass correlation coefficient (ICC). The results yielded an ICC of 0.89 (95% CI: 0.85 – 0.92), indicating excellent measurement consistency and reliability.

#### 4.4.3. Component Alignment

**FIGURE 11:**
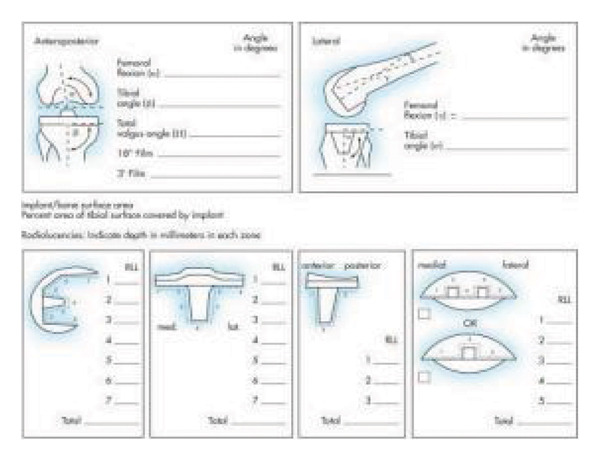
The Knee Society Roentgenographic Evaluation System. Standardized zones and alignment angles (alpha, beta, and sigma angles) utilized for assessing component orientation and monitoring radiolucent lines at the bone–implant interface.

Using the Knee Society Roentgenographic Evaluation System (Figure 2), the following mean values were obtained at the final follow‐up (Figure 11):

 Femoral component (alpha angle): 95.68° (target: 95°–97°, representing 5°–7° valgus relative to the femoral anatomical axis) (Table 3).
 Tibial component (beta angle): 89.27° (target: 90°; neutral/perpendicular to the long axis of the tibia).
 Posterior tibial slope (sigma angle): 6.10° (target: consistent with the natural slope or specific prosthetic design requirements).

#### 4.4.4. Radiographic Stability

No radiographic evidence of component subsidence, radiolucent lines (> 1 mm in any zone), or component migration was noted in any patient at the short‐term follow‐up. These findings suggest that the mechanical reconstruction provided by the “inside‐out” algorithm maintains durable alignment and stable component fixation.

### 4.5. Complications

Despite the severity of Ranawat Type II and III deformities, the complication rate was low:

 Neurological status: Notably, zero cases (0%) of peroneal nerve palsy were recorded. This highlights the safety of the “inside‐out” pie‐crusting technique.
 Periprosthetic fractures: Two cases (6.66%) occurred. One intraoperative femoral fracture was managed with internal fixation; one tibial plateau fracture was bypassed using a modular stem extension.
 Infection: No cases of superficial or deep periprosthetic joint infection (PJI) were observed.

## 5. Discussion

TKA in the valgus knee remains a technically demanding procedure due to the complex constellation of bony deformities and soft‐tissue contractures. The primary finding of this prospective study is that the “inside‐out” release technique, combined with a standard medial parapatellar approach, yields excellent short‐term clinical and radiological outcomes in patients with Ranawat Grade II and III valgus deformities. Our results demonstrated a significant improvement in the mean KSS from 19.63 to 87.17, with 96.6% of patients achieving absolute medial stability.

### 5.1. Surgical Approach and Exposure

There is ongoing debate regarding the optimal surgical approach for the valgus knee. While Keblish [8] and Whiteside [9] advocated for the lateral parapatellar approach to directly address the contracted structures, we exclusively utilized the medial parapatellar approach. We found that this approach provided excellent exposure and allowed for sufficient lateral soft‐tissue release from within the joint (“inside‐out”). This aligns with the findings of Ranawat et al. [1]. The medial approach is particularly advantageous in cases with associated flexion deformity, which was present in 28 of our 30 knees (93.3%), as it facilitates easier access for posterior capsular release.

### 5.2. Soft‐Tissue Balancing Strategy

The crux of success in valgus TKA is the method of soft‐tissue release. Our protocol followed a sequential “inside‐out” release, starting with PCL sacrifice and PLC release, followed by controlled “pie‐crusting” of the ITB.

Our technique mirrors that of Ranawat et al. [1]. In contrast, Whiteside [9] and Krackow et al. [2] described releasing the LCL and popliteus tendon earlier in the sequence. We postulate that preserving the LCL contributes to better midrange stability. Our data support this, as 96.6% of knees achieved absolute medial stability postoperatively.

### 5.3. Comparison With Novel Robotic Technology

In recent years, the emergence of robotic‐assisted TKA (RA‐TKA) has shifted the focus toward “functional alignment.” A notable study by Redmond et al. [10] evaluated the use of robotic platforms specifically in fixed valgus deformities. They found that robotic assistance allowed for more precise bone cuts, which reduced the need for extensive soft‐tissue releases compared to traditional manual methods.

While our manual algorithm aims for a neutral mechanical axis, modern alignment philosophies are gaining traction. Batailler et al. [11] recently highlighted that adopting alternative strategies, such as kinematic or adjusted mechanical alignment, may optimize clinical outcomes by better respecting the patient’s individual anatomy and reducing the necessity for extensive soft‐tissue releases in valgus cases.

The role of technology in managing the valgus knee has been further refined with robotic platforms. Gregori et al. [12] demonstrated that achieving functional alignment through robotic assistance can result in excellent short‐term outcomes with minimal to no soft‐tissue release. Although our study utilized a manual “inside‐out” release, our results match the safety profile of these robotic series, particularly regarding the prevention of peroneal nerve palsy [12, 14].

However, despite the high precision of RA‐TKA, our manual “inside‐out” algorithm achieved a 0% peroneal nerve palsy rate, and comparable safety outcomes were noted relative to reported RA‐TKA. This suggests that while robotics offers advanced planning, a disciplined manual technique remains a highly effective and cost‐efficient alternative, particularly in surgical environments where robotic technology is not yet accessible.

A detailed comparison of our clinical outcomes and complication rates with the international literature is summarized in Table 4.

**TABLE 4:** Comparison of results with international literature.

Reference	Surgical technique	Implant selection	Clinical result (KSS)	Nerve palsy (%)
Current study	Manual inside‐out	70% PS/30% CCK	19.6–87.2	0
Ranawat et al. [1]	Manual inside‐out	100% PS	30.0–93.0	0
Redmond et al. [10]	Robotic‐assisted	PS/CCK	45.0–92.0	0
Apostolopoulos et al. [13]	Lateral + TTO	79% PS/21% CCK	44.0–91.0	0
Krackow et al. [2]	Manual gap balancing	CR	34.4–87.6	3

### 5.4. Implant Constraint Selection

A major challenge in valgus TKA is determining the necessary level of implant constraint. We required a CCK prosthesis in 9 knees (30%), primarily for Grade III deformities with medial incompetence. This rate is higher than that reported by Ranawat et al. [1] (who used PS implants only) but aligns more closely with Apostolopoulos et al. [13], who advocated for CCK use in deformities exceeding 20° to prevent residual instability. We believe that liberal use of constraint is preferable to leaving a knee unstable in elderly patients.

### 5.5. Complications and Safety

The absence of peroneal nerve palsy in our series (0%) suggests that the “inside‐out” algorithm, specifically the incremental pie‐crusting within a 1‐cm safety zone, is a reliable protector of the nerve. While traditional series by Krackow et al. [2] reported palsy rates up to 3% in fixed valgus deformities, our controlled lengthening technique allowed for gradual correction without excessive traction on the CPN. However, we acknowledge that our 95% CI upper limit (11.6%) indicates that a larger multicenter study would be necessary to confirm if this technique truly reduces the risk below the established literature standards.

However, we encountered two intraoperative periprosthetic fractures (6.6%), highlighting the need for caution during trial reduction in osteoporotic bone.

## 6. Limitations

Study design: This was a nonrandomized, prospective study with a relatively small cohort (*n* = 30).

A 12‐month follow‐up is sufficient for assessing early stability and neurological safety but is insufficient for determining long‐term implant survival or late‐onset instability.

Single‐center experience: Findings reflect the expertise of a specialized team and may not be fully generalizable to the broader community.

## 7. Conclusion

The “inside‐out” release technique via a medial parapatellar approach is a safe and reproducible method for correcting fixed valgus deformity. It allows for precise gap balancing while minimizing the risk of peroneal nerve injury. While standard PS implants are sufficient for most cases, surgeons must be prepared to utilize constrained (CCK) implants in approximately one‐third of cases where medial soft‐tissue incompetence prevents adequate stability.

## Author Contributions

Medhat Mohamed Hassan: conceptualization, methodology, and writing–original draft; Molham Mahmood Mohammad: supervision and validation; Khalid Mohamed Abdelhaleem Hafez: data curation; and Alaa El‐din Mohy El‐din Soliman: formal analysis and visualization.

## Funding

No funding was received for this manuscript.

## Conflicts of Interest

The authors declare no conflicts of interest.

## Data Availability

The datasets used and analyzed during the current study are available from the corresponding author on reasonable request.
